# An all-optical modulation method in sub-micron scale

**DOI:** 10.1038/srep09206

**Published:** 2015-03-17

**Authors:** Longzhi Yang, Chongyang Pei, Ao Shen, Changyun Zhao, Yan Li, Xia Li, Hui Yu, Yubo Li, Xiaoqing Jiang, Jianyi Yang

**Affiliations:** 1 Department of Information Science and Electronics Engineering, Zhejiang University, Hangzhou 310027, China; 2 Cyrus Tang Center for Sensor Materials and Applications, Zhejiang University, Hangzhou 310027, China

## Abstract

We report a theoretical study showing that by utilizing the illumination of an external laser, the Surface Plasmon Polaritons (SPP) signals on the graphene sheet can be modulated in the sub-micron scale. The SPP wave can propagate along the graphene in the middle infrared range when the graphene is properly doped. Graphene's carrier density can be modified by a visible laser when the graphene sheet is exfoliated on the hydrophilic SiO_2_/Si substrate, which yields an all-optical way to control the graphene's doping level. Consequently, the external laser beam can control the propagation of the graphene SPP between the ON and OFF status. This all-optical modulation effect is still obvious when the spot size of the external laser is reduced to 400 nm while the modulation depth is as high as 114.7 dB/μm.

The development of information technology requires the highly integrated signal processing circuits. Gradually, the evolution of signal processing systems was mainly separated into two directions, the electronic one and the optical one. The all-electronic processing technique is quite mature in now days and the feature size of the most advanced devices has been reduced to 14 nm according to Intel, which means the all-electronic signal processing circuits can be ultra-compact^1^. However, considering the bottleneck of the interconnection speed as well as the enormous power consumption of the all-electronic approach, the optical modulation method is gaining more and more attractions during the past decade.

As the carrier of the optical signals, the waveguide plays an important role in optical circuits. Silicon optical waveguides can be used as modulators to reach the speed as 100 GHz, but the thickness of the waveguide is as large as hundreds of nanometers^2^. In order to reduce the optical mode size and make a better confinement of the light energy, the Surface Plasmon Polaritons (SPP) wave is brought into use. Some recent SPP modulators can reduce the mode area to about 100 × 100 square nanometers^3^. Furthermore, by introducing the two-dimensional material, graphene, the confinement of SPP is even enhanced and the penetration depth of the graphene SPP is reduced to tens of nanometers^4^,^5^,^6^,^7^,^8^. Even though the optical momentum of the graphene SPP is quite different from that of the light in the free space, some experiments have successfully proved that the graphene SPP could be excited^9^,^10^. Therefore, the graphene SPP modulator is worth being investigated although most of the proposed graphene modulators are based on the silicon waveguides and they are modulated by the electronic methods^2^,^11^,^12^,^13^,^14^,^15^. The electro-opto modulators cannot meet the requirement to construct the all-optical interconnection system or build the all-optical signal processing circuits. For the purpose of making an ultra-compact and all-optical modulator, it is necessary to find out a new way to control the graphene SPP only with the external light beam.

## Results

### Structural description of the all-optical SPP modulator

**Figure 1:**
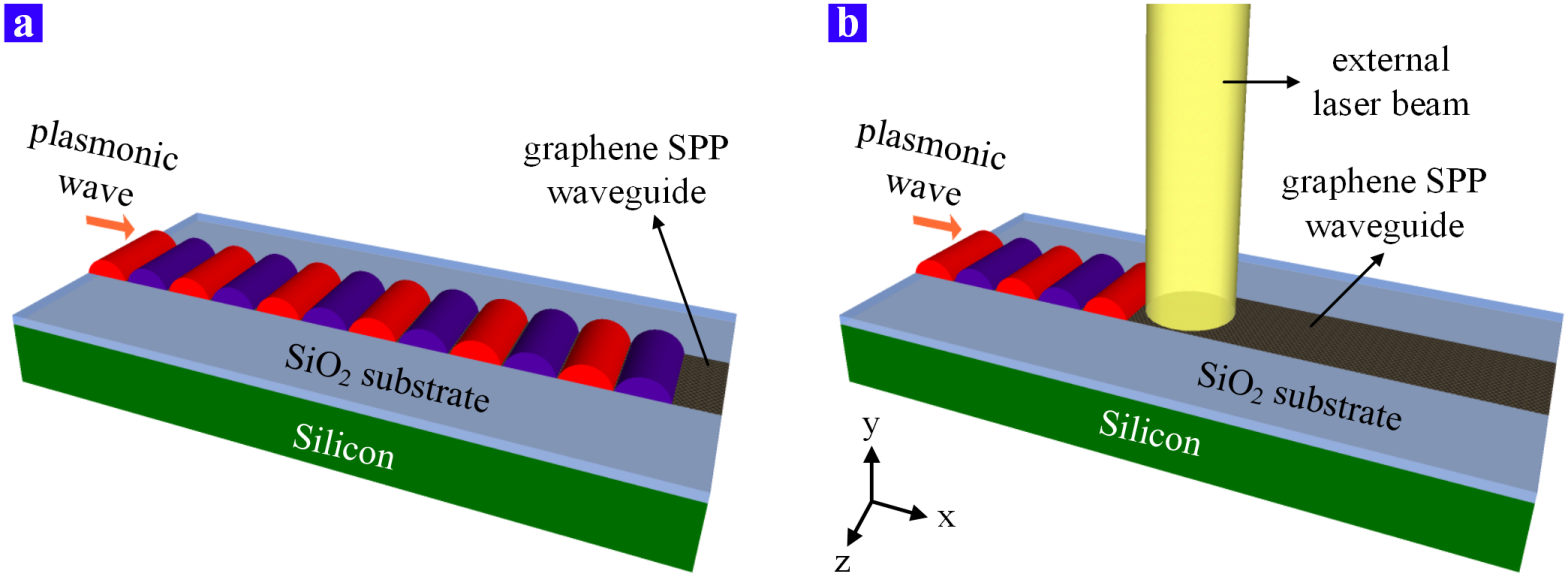
The schematic diagram of the modulator's structure. A piece of graphene is exfoliated on the SiO_2_/Si substrate and the SPP wave propagates from the left side of the graphene. (a) Without the external illumination, the plasmonic wave propagates directly to the right side along the graphene sheet. (b) An external laser beam illuminates onto the graphene so that the propagation of the plasmonic wave is blocked.

Here we theoretically propose an all-optical modulation method for the SPP wave within the size of hundreds of nanometers. This modulator is based on the surface plasmonic wave, which is supported by the doped graphene sheet. The excitation of the graphene SPP has been discussed in lots of papers^9^,^10^,^16^. In order to change the property of graphene by the external laser, a proper substrate material is necessary. For example, we can exfoliate the graphene sheet on a hydrophilic SiO_2_/Si substrate and the initial status of the graphene is p-doped^17^. The thickness of the Si layer is 500 μm while the SiO_2_ layer is as thick as 290 nm. The SPP wave can propagate along such a graphene waveguide since its permittivity is suitable, which is demonstrated as Fig. 1(a). However, the support for the SPP wave is collapsed once if an external laser beam is illuminated onto the graphene sheet as Fig. 1(b) shows. As a result, this proposed all-optical SPP modulator has the ability to realize the OOK (On-Off Keying) modulation scheme.

### Physical mechanism of the modulation method

**Figure 2:**
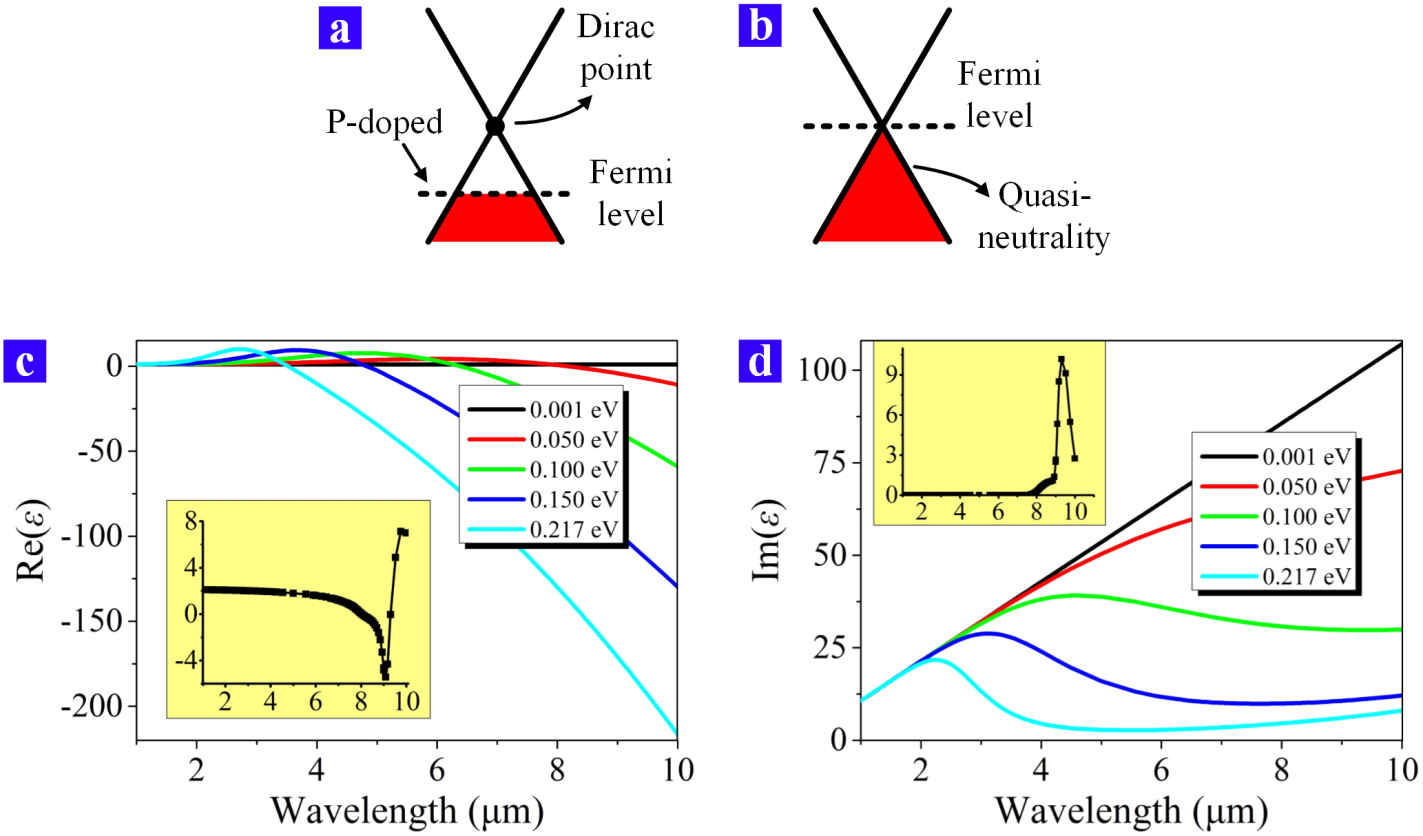
The fundamental properties of graphene. (a) and (b) demonstrate the energy band structures of the electrons in graphene. (a) Without the illumination of the external laser, the graphene is at the p-doped status and the Fermi energy is lower than the Dirac point. (b) Being illuminated, the graphene is at the quasi-neutral status and the Fermi energy is as the same level as the Dirac point. (c) The main panel: the real part of the graphene's permittivity varies against the wavelength. Those five curves correspond to five different chemical potentials of graphene. The insert diagram: the real part of the permittivity of SiO_2_. (d) The main panel shows the imaginary part of the graphene's permittivity while the insert demonstrates Im(*ε*) of SiO_2_.

Graphene is a special two-dimensional material since its permittivity can be obviously changed when its chemical potential is varied. The chemical potential can be treated as the same value as the Fermi energy for the room temperature^4^. Different kinds of approaches can change the graphene's chemical potential, such as the electric gate voltage^12^,^13^, replacing the substrate and the optical irradiation^17^,^18^. The Fermi energy of the graphene, which is exfoliated on the hydrophilic SiO_2_/Si substrate, is lower than the Dirac point as Fig. 2(a) shows^17^. Meanwhile, its Fermi energy can be directly deduced from its carrier density^19^. The initial carrier density of graphene is *n_s_* ≈ 4 × 10^12^ cm^−2^ according to Ref. 17, which means the initial chemical potential is *μ_c_* ≈ 0.217 eV. By bringing in an external visible laser illuminating on the graphene and gradually increasing the laser's power, the graphene is changed from the p-doped status to the light p-doped status, and finally to the quasi-neutral status. The wavelength of the external laser is 532 nm and a laser power as large as 0.6 mW can rise the graphene's Fermi energy to the Dirac point, which is shown as Fig. 2(b). The quasi-neutral status means the carrier density is close to zero and the chemical potential can be estimated as *μ_c_* = 0.001 eV.

Within a certain range of wavelength, the TM polarized wave can be supported by a doped graphene once if the imaginary part of the graphene's optical conductivity, Im(*σ*), is positive^20^. Conversely, the TM mode is not supported when the graphene is changed from the doped status to the quasi-neutral status, which leads to Im(*σ*) < 0. For the purpose of investigating the mode property of the SPP wave on the graphene, it is necessary to utilize the relation between the graphene's conductivity (*σ*) and the chemical potential (*μ_c_*), which is illustrated as the Kubo formulas^21^. In order to construct the simulation model, the equivalent surface permittivity (*ε*) of graphene should be obtained, so that its conductivity (*σ*) and thickness (Δ) are taken into the calculation^6^. Δ is assumed to be 0.34 nm since it is a one-atom thick material. Graphene is anisotropic and the electric field cannot excite any current in the perpendicular direction so that its perpendicular permittvity should be 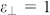.

From the analysis above, the graphene's permittivity is dispersive with different wavelengths and different chemical potentials. Since the external laser can change the graphene's carrier density from 4 × 10^12^ cm^−2^ to nearly zero, the surface permittvity of the graphene should be studied within 0.001 eV ≤ *μ_c_* ≤ 0.217 eV. Fig. 2(c) and (d) demonstrate the real part and imaginary part of the graphene's permittvity for the infrared wavelength. For the real part, Re(*ε*) is almost zero in the infrared range when the graphene is in the quasi-neutral status (*μ_c_* = 0.001 eV). As Fig. 2(c) shows, the value of −Re(*ε*) becomes larger as the doping level of the graphene becomes higher. So, the doped graphene plays the role as a thin layer of metal and it forms the Insulator-Metal-Insulator (IMI) structure together with the air and the SiO_2_/Si substrate. In order to give a better support for the SPP mode, the value of −Re(*ε*) needs to be larger so that the highly doped graphene should be chosen to form the IMI structure. Meanwhile, the loss of the SPP waveguide is another important parameter to be concerned, which is indicated by the imaginary part of the graphene's permittivity, Im(*ε*). Fig. 2(d) demonstrates that Im(*ε*) of the quasi-neutral graphene is always larger than that of the doped graphene, which means the propagation distance of the SPP is longer when the graphene is doped in a higher level. Within the range of 0.001 eV ≤ *μ_c_* ≤ 0.217 eV, we can draw the conclusion that the graphene SPP waveguide is in the ON status when the graphene's chemical potential is *μ_c_* = 0.217 eV while it is in the OFF status when *μ_c_* = 0.001 eV.

The permittivity of the air is always 1 for any range of wavelength. Apart from the dispersive permittvity of the graphene, the dispersive property of the substrate material is also necessary to be taken into consideration. The thickness of the SiO_2_ layer is 290 nm, which is much larger than the lateral decay length of the graphene SPP so that we only consider about the dispersion of the SiO_2_ material in the simulation. The data of the silicon dioxide (glass) is obtained from Ref. 22. The real and imaginary part of its permittvity are shown as the insert diagrams in Fig. 2(c) and Fig. 2(d), respectively. The mode energy of the SPP wave in the IMI structure is mostly distributed outside the metal part so that the SPP wave is quite sensitive to the lossing property of the insulator parts. The SiO_2_ material is nearly lossless within 1 μm ≤ *λ* ≤ 7.634 μm, which oppositely indicates the SPP wave propagating along the graphene sheet may suffer from a severe loss when *λ* is larger than 7.634 μm.

**Figure 3:**
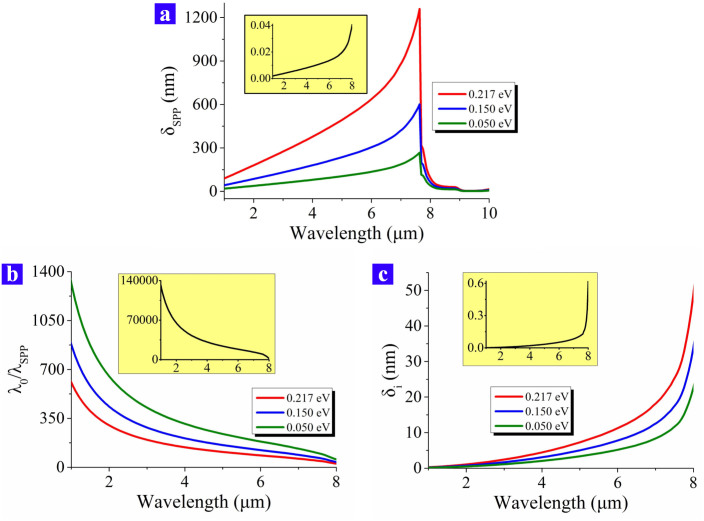
The properties of the SPP wave when it is propagating along the graphene sheet. (a) The propagation distance of the SPP wave varies against the source wavelength. (b) This is the ratio between the source wavelength *λ*_0_ and the SPP wavelength *λ_SPP_*. The variation of the ratio is against the source wavelength. (c) The lateral penetration depth of the SPP wave varies against the source wavelength. Different chemical potentials have different influence on *δ_SPP_*, *λ*_0_/*λ_SPP_* and *δ_i_*. The difference is shown by the red, blue and green curves in (a), (b) and (c). The three colorful curves respectively correspond to *μ_c_* = 0.217 eV, 0.150 eV and 0.050 eV, while the curve in the insert diagrams correspond to *μ_c_* = 0.001 eV.

One of the key parameter of the graphene's SPP wave is the wave vector (*k_SPP_*), which can be influenced by the chemical potential (*μ_c_*), the permittivity of the SiO_2_ substrate (*ε_sub_*) and the wavelength in free space (*λ*_0_) according to Ref. 4. Here we use the permittivity of SiO_2_, which is shown in Fig. 2(c) and (d). The difference between the ON and the OFF status of the SPP wave is determined by its propagatin distance, which can be expressed as *δ_SPP_* = 1/2Im(*k_SPP_*). The propagation distance of the SPP wave is demonstrated in Fig. 3(a) for 4 sorts of chemical potentials. As the source wavelength (*λ*_0_) increased, *δ_SPP_* becomes larger until *λ*_0_ = 7.634 μm. For *λ*_0_ > 7.634 μm, *δ_SPP_* drops very quickly because the SPP energy penetrates inside the SiO_2_ substrate and it is lost severely due to the large Im(*ε*) of SiO_2_. The red, blue and green curves in Fig. 3(a) demonstrate that *δ_SPP_* is as large as hundreds of nanometers when the graphene is doped. However, when the graphene is illuminated by the external laser and be tuned back to the quasi-neutral status, *δ_SPP_* is smaller than 1 nanometer, which is demonstrated as the insert diagram in Fig. 3(a). In other words, the SPP wave is definitely not able to propagate along the graphene sheet when the graphene's chemical potential is close to zero. Furthermore, the wavelength of the SPP wave (*λ_SPP_*) is much smaller than *λ*_0_ since *λ_SPP_* = 2*π*/Re(*k_SPP_*). From the results shown in Fig. 3(b), *λ*_0_ is hundreds of times larger than *λ_SPP_* when the graphene is doped. As the insert of Fig. 3(b) shows, *λ*_0_ is more than ten thousands times larger than *λ_SPP_* when the external laser makes the graphene be in the quasi-neutral status. Similarly, the lateral penetration depth of the SPP wave (*δ_i_*) shows the same trend since *δ_i_* = *λ_SPP_*/2*π*, which is demonstrated in Fig. 3(c). Both Fig. 3(b) and 3(c) illustrate that graphene has a strong confiment of the SPP wave with the scale of tens of nanometers so that the graphene SPP is appropriate for designing ultra-compact optical devices.

### Modulation effects of the all-optical method

**Figure 4:**
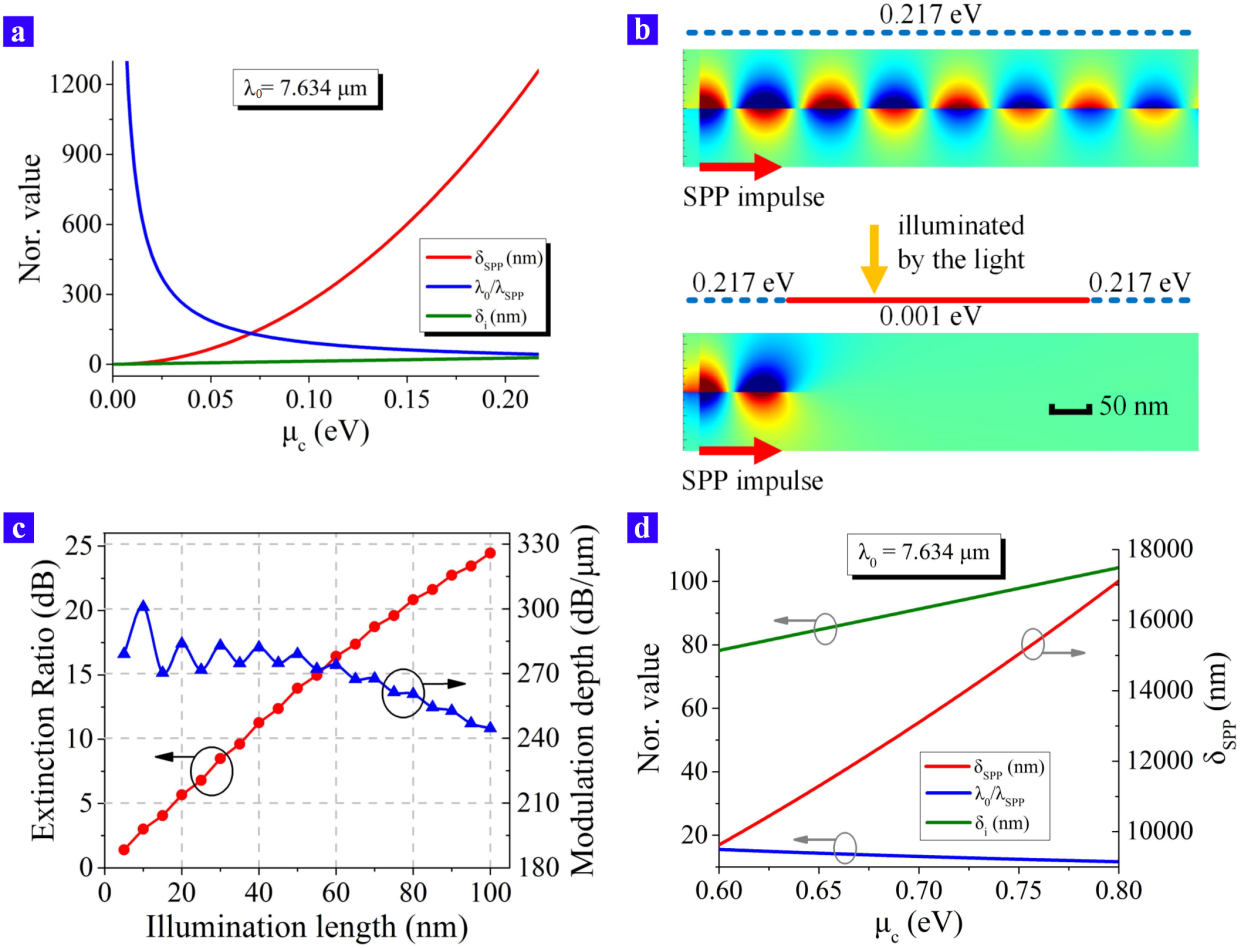
The optical properties and modulation effects of the SPP wave for a certain wavelength *λ*_0_ = 7.634 μm. (a) *δ_SPP_*, *λ*_0_/*λ_SPP_* and *δ_i_* are all changed when *μ_c_* is increased, which are respectively shown by the red, blue and green curves. The unit of *δ_SPP_* and *δ_i_* is nanometer while the ratio *λ*_0_/*λ_SPP_* is dimensionless. (b) The simulation results of the proposed device are shown here. The SPP impulse propagates from the left side towards the right side. The graphene illuminated by the external laser is changed to be *μ_c_* = 0.001 eV while the unilluminated part is *μ_c_* = 0.217 eV. Scale bar, 50 nm. (c) The extinction ratio as well as the modulation depth are both varied against the illumination length of the external laser, which are in the assumption that the laser's spot size is reduced to the nanometer scale. (d) The optical properties of the SPP wave for a certain wavelength *λ*_0_ = 7.634 μm for the fictitiously highly doped graphene, which is on the condition: 0.6 eV < *μ_c_* < 0.8 eV.

To demonstrate the all-optical modulation method clearly, we should make the Extinction Ratio (ER) between the ON and OFF status as big as possible. Fig. 3(a) indicates that the source wavelength should be fixed at *λ*_0_ = 7.634 μm. As far as the SPP wave is concerned, both the propagation distance and the lateral penetration depth are increased while the ratio *λ*_0_/*λ_SPP_* is decreased when *μ_c_* is increased from 0.001 eV to 0.217 eV, which are shown in Fig. 4(a). In order to exhibit the effects of the all-optical modulation, the commercial software “FDTD Solutions” is utilized for the simulation, whose results are demonstrated in Fig. 4(b). In the upper part of Fig. 4(b), a piece of graphene is exfoliated on a hydrophilic SiO_2_/Si substrate and the substrate makes the graphene be doped as *μ_c_* = 0.217 eV. The SPP wave is excited by a source and the impulse propagates from the left side. Since the doped graphene provides a good support for the SPP wave, the impulse propagates along the graphene sheet towards the right side and the modulator shows an ON status. In the lower part of Fig. 4(b), an external laser beam is brought in and it illuminates onto the graphene sheet. The illumination area cannot be too small when the diffraction limit is taken into consideration. Since the wavelength of the external laser is 532 nm, its spot size is set to be 400 nm considering the diffraction limit. After being illuminated, the graphene is changed from the p-doped type to the quasi-neutral type. When the red area is turned to be *μ_c_* = 0.001 eV, that section of graphene can no longer support the SPP wave. As a result, the SPP energy is rapidly absorbed at the illuminated part (see Supplementary section 4). In this condition, the right side of the graphene can receive nearly no SPP energy so that the whole device shows an OFF status. According to the simulation results, the ER is about 45.9 dB for *λ*_0_ = 7.634 μm and it indicates the modulation depth reaches 114.7 dB/μm.

## Discussion

As far as the diffraction limit is concerned, the spot size of the external laser can be only reduced to about half of its wavelength, which means the illumination length can only be reduced to the sub-micron scale. If the spot size of the laser is as small as several nanometers, the proposed SPP modulator may obtain a smaller feature size. Practically, some experimental data demonstrate that the mode size of the light emission can be reduced and break through the diffraction limit by the hybrid plasmonic structures^23^,^24^,^25^,^26^. For the visible wavelength, the CdS-MgF_2_-Ag nano-strcuture even confine the light energy within a feature size of several nanometers to design the sub-diffraction-limited lasers^24^,^25^. As a result, a further investigation is made and the illumination length of the modulator is varied from 5 to 100 nm to study the possibility of modulating the SPP signals in the nanometer scale. Since the illumination length is the effective modulation length, the modulation depth is also varied. The simulation results are demonstrated in Fig. 4(c), which indicates that the ER is larger than 10 dB when the illumination length is longer than 40 nm. It illustrates that such a SPP modulator has the ability to control the light signals within a nanometer scale once if the light confinment enhancement of the hybrid plasmonic structure is utilized to decrease the spot size of the external laser.

Nevertheless, a general and significant problem of the SPP wave also remains in our proposed method: the loss. As the numerical analysis show, the propagation distance of the SPP wave is about 1260 nm for *λ*_0_ = 7.634 μm and *μ_c_* = 0.217 eV, which indicates the device has to be very short. Being limited by the optical doping level, the chemical potential of graphene can just vary from 0 eV to 0.217 eV. If some other optical doping procedures, which can change the chemical potential in a larger range, are utilized, we may gain a longer propagation distance of the SPP wave. For instance, using the irradiation in the UV wavelength or replacing the SiO_2_/Si substrate by some other material may gain a better results. Here an assumption is made that the graphene can be optically doped from 0 eV to 0.8 eV. Then, the properties including *δ_SPP_*, *δ_i_* and *λ*_0_/*λ_SPP_* are demonstrated in Fig. 4(d) as the chemical potential varies. The red curve in Fig. 4(d) tells that if the graphene is highly doped to *μ_c_* = 0.8 eV, the propagation distance can be increased by more than 10 times to 17000 nm, which will effectively benefit the construction of the optical circuits.

In addition, the reason why we choose graphene to be the waveguide of this all-optical modulator is that the graphene's permittivity can be obviously modified by the external laser and Re(*ε*) of the graphene is a negative value. The tunability of the permittivity is of great importance in integrated optics but not only graphene possesses such a property. In the future, researchers can try looking for some material with such a tunability, such as some other two-dimensional or the photosensitive substance, to design the all-optical devices.

In conclusion, by exfoliating the graphene sheet onto a hydrophilic SiO_2_/Si substrate and utilizing an external laser to illuminate a part of the graphene, a modulation method is proposed for the SPP wave with two specialties including all-optical and small feature size. The illumination length is as short as 400 nm and it obtains an extinction raito as high as 45.9 dB, which means the modualtion depth reaches 114.7 dB/μm. The all-opitcal modulation can be realized in some other silicon structures such as the micro-ring and the fiber but the footprint is in the scale of several microns, which cannot be equal in force with the all-electronic modulation. Based on the all-optical modulation of the surface plasmonic wave, our proposed method decreases the feature size into the sub-micron scale with a quite high modulation depth. Such kind of thoughts can be applied for designing the fundemental devices in the all-optical interconnection systems, which may progress the construction of the large-scale integration optical circuits in the future.

## Methods

The simulations are performed in the 2D type with the help of the finite-different time-domain method by using “Lumerical FDTD Solutions”. The effects shown in Fig. 4(b) are the magnetic field distribution in the z direction. The total simulation region is set in the boundary condition as the perfectly matched layers (PML) while the area of the simulation region is 930 nm × 500 nm (see Supplementary Fig. S1-1 online). The width of the source is 250 nm and the SPP mode is manually selected. The whole domain is discretized by using an inhomogeneous rectangle mesh with the maxmium element size of 1 nm × 1 nm. The domain where the graphene exists needs a finer mesh so that the mesh size perpendicular the graphene sheet is 0.17 nm (see Supplementary Fig. S3 online).

## Author Contributions

L.Z.Y. designed the work and wrote the manuscript. C.Y.P. and A.S. provide the optical doping method of the graphene. C.Y.Z. and X.L. discussed about the excitation of the SPP mode on graphene in the simulation. Y.L. and H.Y. performed theoretical analyses and simulations. Y.B.L. and X.Q.J. helped to plot all the figures. J.Y.Y. supervised the project. All authors discussed the results and commented on the manuscript.

## Supplementary Material

Supplementary Information Supplementary information
