# Supplementary material for: An all-optical modulation method in sub-micron scale
Source: Sci Rep. 2015 Mar 17;5:9206. doi: 10.1038/srep09206 (PMC4361842; doi:10.1038/srep09206)
Supplement: Supplementary Information [file srep09206-s1.pdf]

# Supplementary Information

## An all-optical modulation method in sub-micron scale

Longzhi Yang, Chongyang Pei, Ao Shen, Changyun Zhao, Yan Li, Xia Li,

Hui Yu, Yubo Li, Xiaoqing Jiang, and Jianyi Yang

### 1. Simulation model

The simulations are performed by the software “FDTD Solutions”. The gray part in Fig. S1-1 is the $SiO_2$ substrate, whose permittivity is manually input according to Ref. S1. The black part is the air, whose refractive index is 1. The graphene waveguide is between the $SiO_2$ substrate and the air. The surface plasmonic wave is excited by a mode source with a width of 250 nm, which is shown by the purple arrow. A transmission monitor, which records the transmission power of the SPP energy, is set on the right side of the source and their distance is 600 nm. A reflection monitor is set on the left side of source in order to see whether the SPP energy is reflected by the illuminated graphene. A global monitor with the size of 930 nm × 500 nm is put around the graphene waveguide to view how the SPP energy propagates. The magnetic field of the SPP energy is recorded.

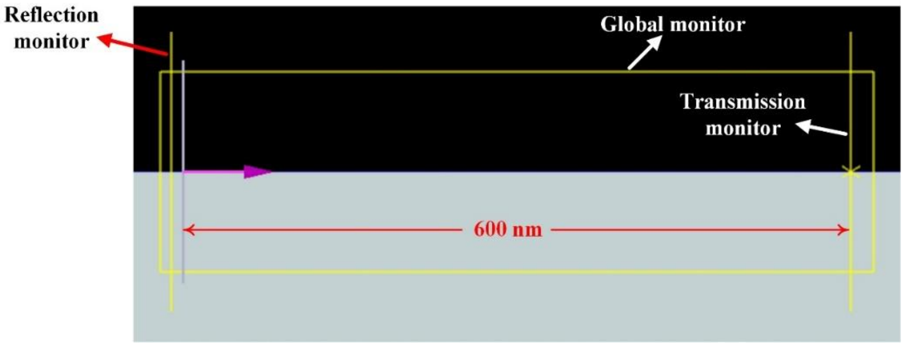


**Figure S1-1. The simulation model of the graphene SPP modulator.**

The geometric coordinate of the simulation model is marked out in Fig. S1-2. The relative position of the graphene and the SPP source are shown in this figure. For the OFF status, the graphene is illuminated on the position 100 nm < $x$ < 500 nm.

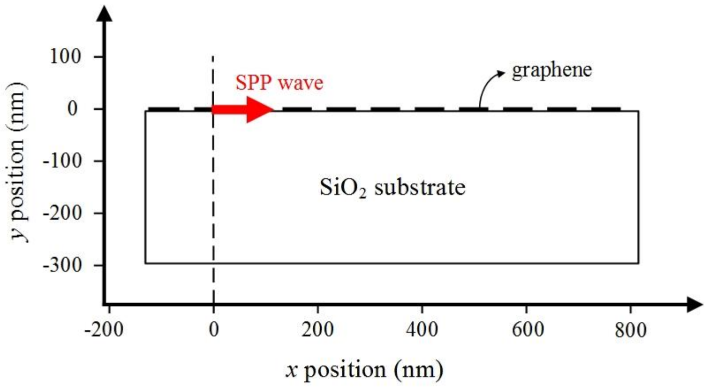


**Figure S1-2. The geometric coordinate of the simulation model.**

## 2. Field distribution of the source mode

The normalized field distribution of the source mode, which is manually selected, is shown in Fig. S2. Such a stable TM mode can propagate along the graphene waveguide. Fig. S2(a) shows that the air, the graphene and the $SiO_2$ substrate form the Insulator-Metal-Insulator structure so that the E field is mainly concentrated beside the graphene layer. The lateral penetration depth of the graphene SPP is only tens of nanometers. Fig. S2(b) shows that the H field distributed in the air part is not symmetric with the one in the $SiO_2$ substrate since the air's permittivity is not the same as the permittivity of $SiO_2$.

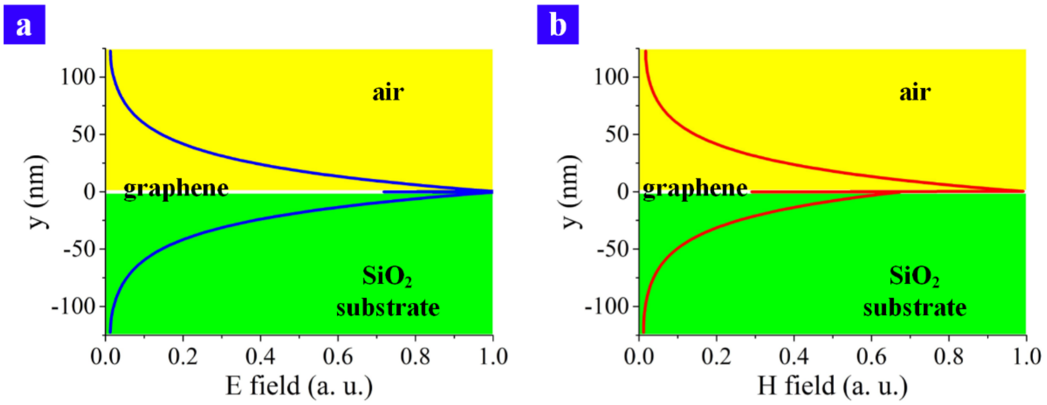


**Figure S2. Field distribution of the source mode.**

## 3. Mesh grid size

This is a detailed view of the mesh grid of the graphene layer. The whole simulation region is partitioned by the inhomogeneous rectangle mesh grids. The graphene sheet is equally partitioned into two layers with the size of 0.17 nm along the y direction for the calculation in all the simulations. The maximum mesh grid size outside the graphene layer is 1 nm along the y direction. For all the materials, the mesh grid size along the x direction is 1 nm.

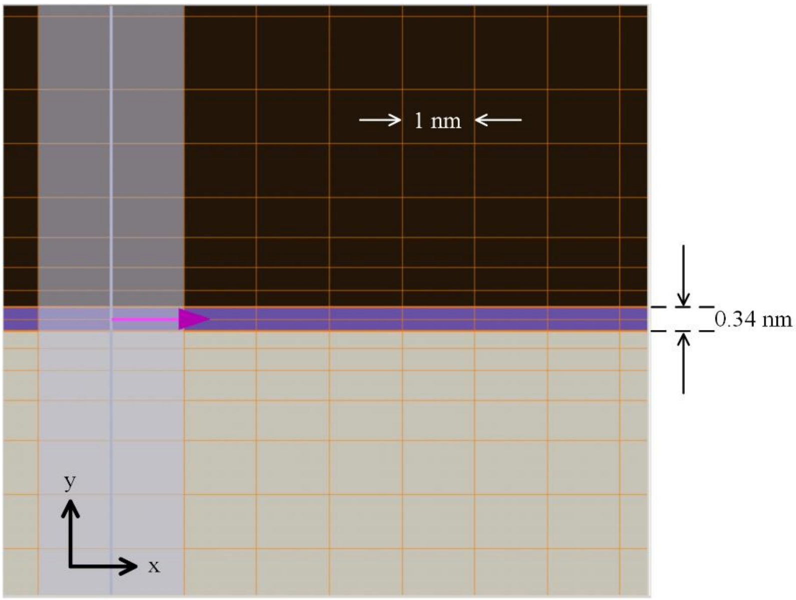


**Figure S3. Mesh grid size in the simulation.**

**4. The propagation and decay of the SPP energy**

When the proposed modulator is in the OFF status, the SPP energy seems to be suddenly disappeared at the illuminated graphene. In order to demonstrate the propagation and decay of the SPP energy, the normalized transmission and power distribution of the light energy are shown as the following part.

① For the ON status:

In this condition, no external laser is illuminating on the graphene. The normalized power of the SPP wave is decreased when it propagates along the graphene waveguide, which is shown in Fig. S4-1. For the position $x < 0$, there is almost no power so that the reflected SPP energy is nearly zero for the ON status.

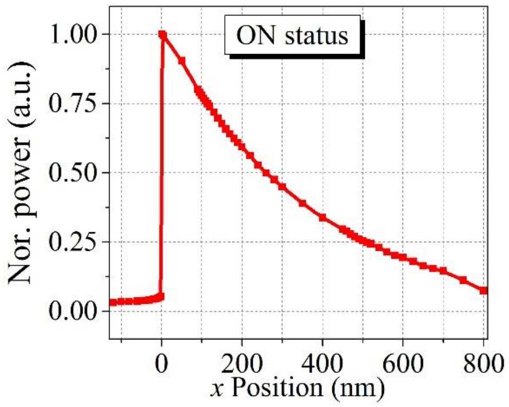


**Figure S4-1. The variation of the SPP energy for the ON status.**

For the purpose of confirming the confinement of the SPP wave, the vertical power distributions are observed on different *x* positions as Fig. S4-2 shows. The light energy is always in the form of the Surface-Plasmon-Polaritons wave but the amplitude is becoming smaller. The SPP mode means the light energy is only distributed around the graphene waveguide. Consequently, the reason for the decrease of the SPP power only comes from the lossy property of the graphene itself for the ON status.

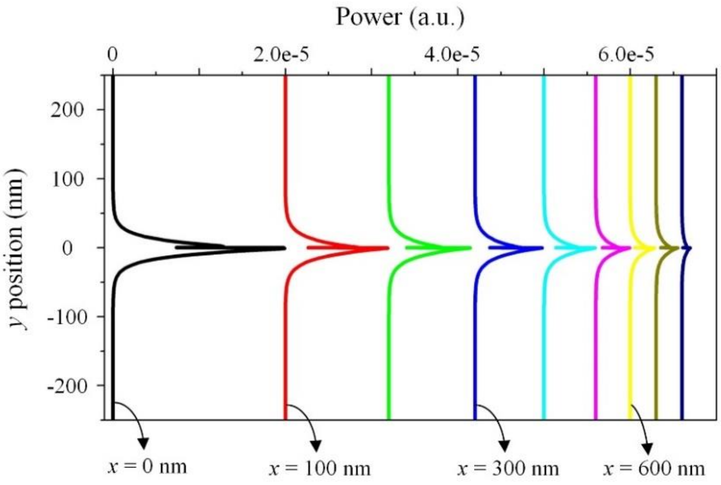


**Figure S4-2. The vertical power distribution on different *x* positions for the ON status.** There are 9 curves in this picture, which respectively refer to $x$ = 0, 100, 200, 300, 400, 500, 600, 700, 800 nm in the simulation.

② For the OFF status:

In this condition, the graphene is illuminated for 100 nm < $x$ < 500 nm. Fig. S4-3 demonstrates the normalized power for the OFF status and it shows that a large amount of SPP energy is reflected. The power on the position $x = 0_+$ nm is p = 0.66 rather than p = 1. This is because the reflected energy counteracts a part of the recorded data. On the position $x = 0_-$ nm, the power is about p = 0.34, which indicates that more than 30% of the SPP energy is reflected. The reason for the reflection comes from the difference of the graphene's permittivity. As for the graphene's permittivity, $\varepsilon = -116.2 + 4.1i$ for $x < 100$ nm. When $x > 100$ nm, the permittivity is changed to be $\varepsilon = 0.93 + 81.7i$. A part of the SPP wave has to be reflected when the permittivity of the waveguide is suddenly changed during the propagation process. Therefore, the reflected energy is the first reason for the decay of the SPP power.

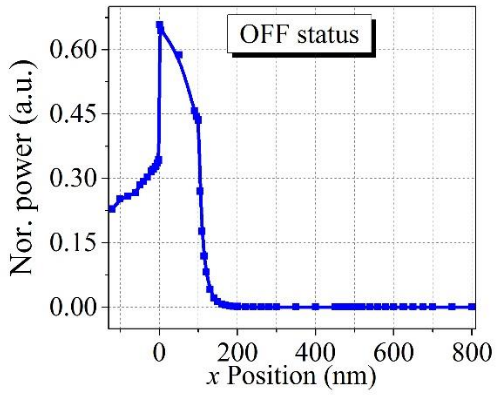


**Figure S4-3. The variation of the SPP energy for the OFF status.**

On the other hand, the 2D power distribution is demonstrated for 90 nm < $x$ < 120 nm with a detailed observation, which is shown in Fig. S4-4. The results demonstrate that the SPP energy is quickly absorbed by the graphene rather than being refracted when $x$ > 100 nm.

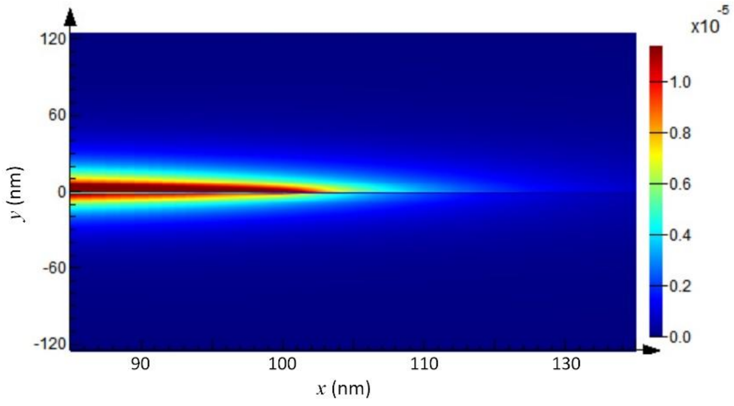


**Figure S4-4. The 2D power distribution for the OFF status for 90 nm < $x$ < 120 nm.**

However, it is necessary to utilize the vertical power distributions of the SPP energy in order to prove that the light energy is mostly confined around the graphene. Fig. S4-5 shows that the shape of the SPP wave still exists even if $x$ > 100 nm. Thus, the light energy is still confined around the graphene for 100 nm < $x$ < 120 nm. It indicates that the light energy does not leak into the air or the substrate when it is propagated along the illuminated graphene for 100 nm < $x$ < 120 nm.

Furthermore, the imaginary part of the graphene's permittivity, Im($\varepsilon$), is the main reason for the decay of the light energy. Im($\varepsilon$) is increased from 4.1 to 81.7 at $x$ = 100 nm, which means the absorption of the graphene is severely increased. Combining the confinement of the SPP energy with the large Im($\varepsilon$) of graphene, these two reasons lead to the consequence that the SPP energy is suddenly disappeared during its propagation for $x$ > 100 nm. In another word, the light energy is quickly absorbed by the illuminated graphene, which is the second reason for the decay of the SPP power.

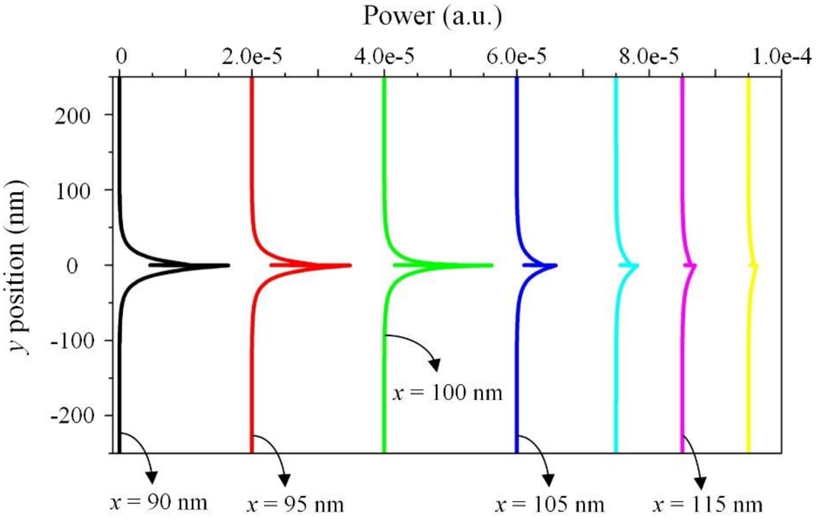


**Figure S4-5. The vertical power distribution on different *x* positions for the OFF status.** There are 7 curves in this picture, which respectively refer to $x$ = 90, 95, 100, 105, 110, 115, 120 nm in the simulation.

### 5. The propagation of the SPP energy for the nanometer modulation

The magnetic fields of the SPP energy are shown in Fig. S5. All these simulations here are based on the assumption that the spot size of the external laser is reduced to the nanometer scale. the magnitudes of the magnetic fields are shown from (a) to (d). The real part values of the magnetic fields in the z direction are shown from (e) to (h). (a) and (e) show the propagation of the SPP when there is no external light illuminating on the graphene. (b) and (f) show the SPP when the illumination length is 5 nm. Since the illumination length is quite short, the propagation of the SPP is only slightly influenced. (c) and (g) show the SPP when the illumination length is 50 nm. The illumination length is long enough so that most of the SPP energy cannot be transmitted to the right side of the modulator. (d) and (h) show the SPP when the illumination length is 100 nm. In this condition, the SPP signals are completely cut off at the illumination section and its extinction ratio is about 25 dB.

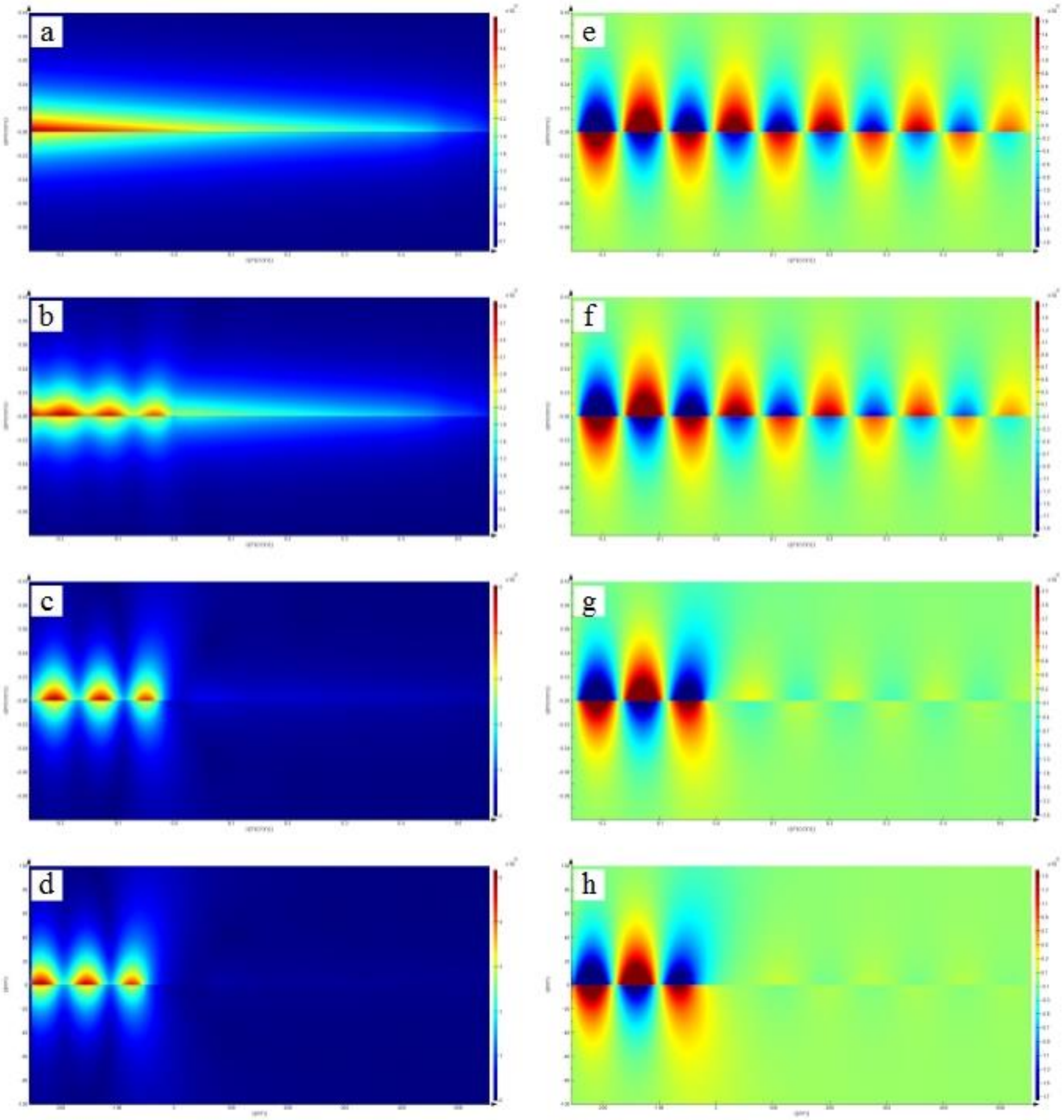


**Figure S5. The propagation of the SPP energy for the nanometer modulation.**

**Reference**

S1. Palik, E. D. Handbook of Optical Constants of Solids [Palik, E. D. (ed.)] [761−762] (Academic press, San Diego, 1998)
